# Robot-assisted laparoscopic myomectomy for FIGO type II sub-mucosal leiomyoma without endometrial injury for a patient with history of miscarriage

**DOI:** 10.4274/jtgga.galenos.2020.2020.0139

**Published:** 2021-02-24

**Authors:** Ayah Hijazi, Youn-Jee Chung, Hee Jin Kang, Jae Yen Song, Hyun Hee Cho, Mee-Ran Kim

**Affiliations:** 1 Department of Obstetrics and Gynecology, Seoul St. Mary’s Fibroid Center Hospital, College of Medicine, The Catholic University of Korea, Seoul, South Korea

**Keywords:** Endometrium, surgical procedures, robotic, fibroid uterus

## Abstract

To introduce a technique for robot-assisted laparoscopic myomectomy for FIGO type II sub-mucosal leiomyoma with >50% myometrial extension, without endometrial injury. A narrated video demonstration of our technique has been provided. Our patient was a 35-year-old, gravida 1, para 0 woman with secondary infertility. She had been married for three years. She complained of heavy menstrual bleeding and severe dysmenorrhea with a pain score of 10 on visual analogue scale (VAS). Surgery was done after thorough counseling and an informed consent was obtained. Institutional Review Board number: KC17OESI0375, approval date: 21.09.2018. Several steps can be taken to help prevent endometrial injury, and these include: (1) proper preoperative imaging to plan surgery; (2) use of intraoperative ultrasound to determine best location of incision; (3) use of a “cold cut” technique with monopolar curved scissors without energy to avoid obscuring the border between the leiomyoma and the endometrium; (4) careful millimeter by millimeter dissection; (5) use of diluted indigo carmine to aid delineation of the endometrial cavity during dissection. The patient had a normal post-operative course. On follow-up her VAS pain score was 0. Transvaginal ultrasound repeated four months postoperatively showed normalization of uterine anatomy and endometrial contour. Robot-assisted laparoscopic myomectomy may be an option to preserve fertility and minimize endometrial injury. This surgical method allows complete removal of large sub-mucosal leiomyomas in one session with exact suturing.

## Introduction

Uterine leiomyomas are the most common benign gynecologic tumors, with an estimated 70-80% of reproductive-aged women having leiomyomas (1). Most patients are asymptomatic but around 30% to 40% present with symptoms. Symptoms depend on location, size and number of leiomyomas (2) and include heavy menstrual bleeding, pain, pressure symptoms, infertility and recurrent pregnancy loss (3).

Around 5-10% of patients with infertility are found to have leiomyomas, with 1-2.4% having them as the only finding. Patients with sub-mucous myomas were found to have a lower clinical pregnancy rate, implantation rate, and ongoing pregnancy and live birth rate, with a significantly higher spontaneous abortion rate (4). Casini et al. (5) found that pregnancy rates were higher after myomectomy in women with sub-mucosal leiomyomas compared with expectant management.

In this video (Video 1) we present our technique for management of type II leiomyoma, in a 35-year old woman with a history of infertility. She had a history of one first trimester miscarriage and was complaining of heavy menstrual bleeding and severe dysmenorrhea. Management options were discussed, and it was decided to opt for robot-assisted laparoscopic removal as, in her case, hysteroscopic removal may have resulted in a significant destruction of the enodometrial surface. Robot-assisted laparoscopic removal can also be beneficial when the leiomyoma is large and may need more than one operation for complete removal. A robot-assisted technique is also a good option when the distance between the leiomyoma and the serosa is small, in order to avoid the risk of perforation.

**Figure 1:**
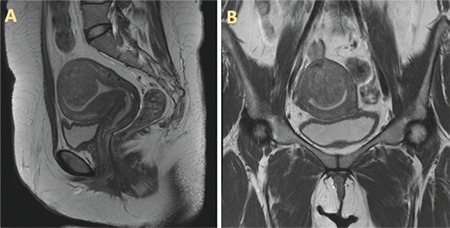
Pelvic magnetic resonance imaging (MRI). (A) Sagittal and (B) coronal pelvic MRI showed a sub-mucosal, posterior fundal uterine leiomyoma measuring 4x4x4.5 cm

**Figure 2:**
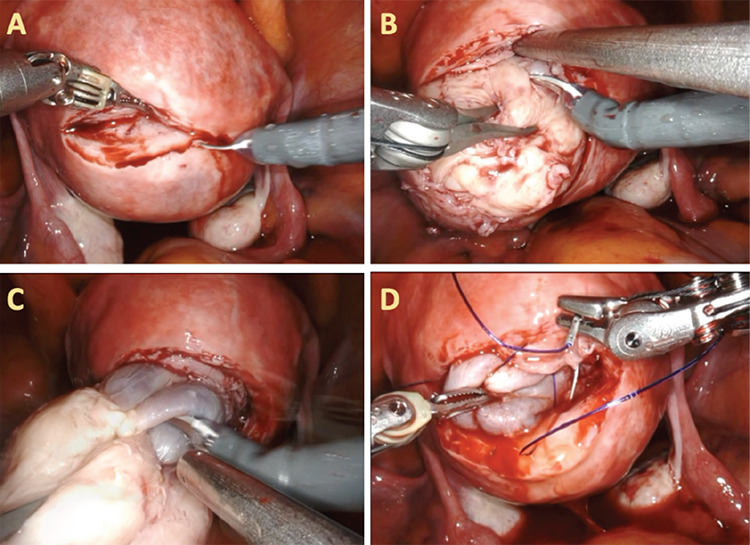
Surgical procedure. Monopolar curved scissors without electrocauterization (also called “cold-cut”), was used to make a transverse incision along the uterine wall overlying the leiomyoma (A). A tenaculum forceps was then used to provide traction while sharp and mechanical dissection was continued (B). To ascertain endometrial integrity and to aid in dissection a diluted indigo carmine solution was injected into the endometrial cavity through the RUMI uterine manipulator (C). After enucleation of the leiomyoma, the defect was sutured in two layers (D) Figure 1. Pelvic magnetic resonance imaging (MRI). (A) Sagittal and (B) coronal pelvic MRI showed a sub-mucosal, posterior fundal uterine leiomyoma measuring 4x4x4.5 cm

A clinical aim was to keep the endometrium intact and this was achieved through several steps, starting with proper preoperative imaging (Figure 1) and planning. In our opinion it is also helpful to use a transvaginal ultrasound to determine the best location for the incision. With the use of “cold cut” technique, using a monopolar curved scissors without energy, and by careful millimeter-by-millimeter dissection, removal of the leiomyoma was accomplished with minimal damage to the adjacent myometrium. Diluted indigo carmine can aid in dissection as it delineates the cavity and it can also aid in suturing the endometrium, in case of injury (Figure 2).

**Figure 3:**
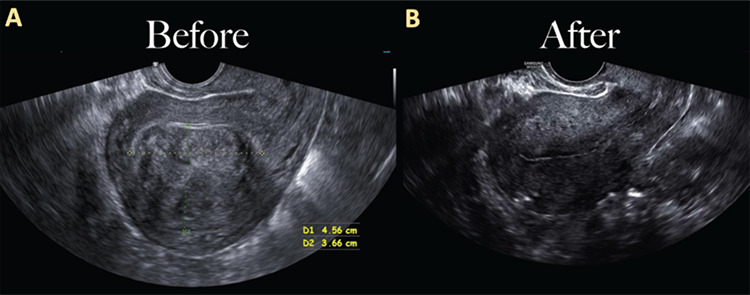
Transvaginal ultrasound of the uterus. Follow up transvaginal ultrasound (B) done 4 months postoperatively showed normalization of uterine anatomy and endometrial contour, compared to the preoperative ultrasound (A)

After removal of the leiomyoma, the defect was closed in two layers and the leiomyoma was then removed via contained morcellation. The patient was followed up with an ultrasound four months after surgery, which showed normalization of the uterine anatomy and endometrial contour (Figure 3).

Robot-assisted laparoscopic myomectomy may be an option to preserve fertility and minimize endometrial injury. This surgical method allows complete removal of large sub-mucosal leiomyomas in one session with exact suturing.

**https://www.doi.org/10.4274/jtgga.galenos.2020.2020.0139.video1**
